# Key Genes and Biochemical Networks in Various Brain Regions Affected in Alzheimer’s Disease

**DOI:** 10.3390/cells11060987

**Published:** 2022-03-14

**Authors:** Morteza Abyadeh, Nahid Tofigh, Saeedeh Hosseinian, Mafruha Hasan, Ardeshir Amirkhani, Matthew J. Fitzhenry, Veer Gupta, Nitin Chitranshi, Ghasem H. Salekdeh, Paul A. Haynes, Vivek Gupta, Koorosh Shahpasand, Mehdi Mirzaei

**Affiliations:** 1 Faculty of Basic Sciences and Advanced Medical Technologies, Royan Institute for Stem Cell Biology and Technology, ACECR, Tehran 1665659911, Iran; mabyadeh94@gmail.com; 2 Department of Brain and Cognitive Sciences, Cell Science Research Center, Royan Institute for Stem Cell Biology and Technology, ACECR, Tehran 1665659911, Iran; tofigh467@yahoo.com; 3 School of Biological and Behavioural Sciences, Queen Mary University of London, London E1 4NS, UK; s.hosseinian@qmul.ac.uk; 4 School of Life and Environmental Sciences, University of Sydney, Sydney, NSW 2006, Australia; mafruha.hasan@sydney.edu.au; 5 Australian Proteome Analysis Facility, Macquarie University, Sydney, NSW 2109, Australia; ardeshir.amirkhani@mq.edu.au (A.A.); matthew.fitzhenry@mq.edu.au (M.J.F.); 6 School of Medicine, Deakin University, Geelong, VIC 2600, Australia; veer.gupta@deakin.edu.au; 7 Department of Clinical Medicine, Faculty of Medicine, Health and Human Sciences, Macquarie Medical School, Macquarie University, Macquarie Park, North Ryde, Sydney, NSW 2109, Australia; nitin.chitranshi@mq.edu.au (N.C.); vivek.gupta@mq.edu.au (V.G.); 8 School of Natural Sciences, Macquarie University, Macquarie Park, NSW 2109, Australia; hsalekdeh@yahoo.com (G.H.S.); paul.haynes@mq.edu.au (P.A.H.); 9 Biomolecular Discovery Research Centre, Macquarie University, Sydney, NSW 2109, Australia

**Keywords:** Alzheimer’s disease, GABAergic synapse pathway, retrograde endocannabinoid signaling, differentially expressed genes

## Abstract

Alzheimer’s disease (AD) is one of the most complicated progressive neurodegenerative brain disorders, affecting millions of people around the world. Ageing remains one of the strongest risk factors associated with the disease and the increasing trend of the ageing population globally has significantly increased the pressure on healthcare systems worldwide. The pathogenesis of AD is being extensively investigated, yet several unknown key components remain. Therefore, we aimed to extract new knowledge from existing data. Ten gene expression datasets from different brain regions including the hippocampus, cerebellum, entorhinal, frontal and temporal cortices of 820 AD cases and 626 healthy controls were analyzed using the robust rank aggregation (RRA) method. Our results returned 1713 robust differentially expressed genes (DEGs) between five brain regions of AD cases and healthy controls. Subsequent analysis revealed pathways that were altered in each brain region, of which the GABAergic synapse pathway and the retrograde endocannabinoid signaling pathway were shared between all AD affected brain regions except the cerebellum, which is relatively less sensitive to the effects of AD. Furthermore, we obtained common robust DEGs between these two pathways and predicted three miRNAs as potential candidates targeting these genes; hsa-mir-17-5p, hsa-mir-106a-5p and hsa-mir-373-3p. Three transcription factors (TFs) were also identified as the potential upstream regulators of the robust DEGs; ELK-1, GATA1 and GATA2. Our results provide the foundation for further research investigating the role of these pathways in AD pathogenesis, and potential application of these miRNAs and TFs as therapeutic and diagnostic targets.

## 1. Introduction

Alzheimer’s disease (AD) is the leading cause of dementia, affecting between 70 to 80% of older adults with dementia [1]. Currently, over 50 million people are living with the disease worldwide, and this number is estimated to rise to 150 million in 2050, exacerbating an already constrained healthcare system unless preventive strategies are implemented [2,3]. AD is characterized by initial memory loss and learning impairment, followed by cognitive dysfunction. The disability progresses significantly throughout the disease course, culminating in death within 5–12 years of the onset of symptoms [3,4]. The current treatments only provide symptomatic relief without mitigating disease progression. Thus, there are a growing number of studies focusing on potential therapeutic agents to combat AD more directly [5,6,7]. Most of these studies are focused on two main pathological hallmarks of AD: senile plaques (SPs) composed of amyloid beta (Aβ) peptides; and neurofibrillary tangles (NFTs) composed of hyperphosphorylated tau proteins [3,4,5,8]. Although results of clinical trials have been underwhelming for the past 25 years, recently an anti-amyloid β antibody, Aducanumab, received an accelerated FDA approval, requiring further clinical trials to confirm the estimated efficacy [1]. Repeated failure in clinical trials has challenged our understanding of this multifactorial disease, leading to recent studies concentrating on advancing our knowledge of the underlying mechanisms of AD pathogenesis to find druggable targets.

High-throughput ‘omics’-based research including genomics, transcriptomics and proteomics has made a significant contribution to our current understanding of AD [9,10,11]. However, currently the biological data are generated at a higher pace than they are being interpreted. Thus, there is an urgent need to summarize and extract new knowledge from the existing data. We have used meta-analysis to summarize and extract the most reliable data from existing results of multiple studies, taking advantage of the increased statistical power of larger combined sample sizes [12,13]. Several meta-analyses have been performed on microarray gene expression datasets of different brain regions to identify altered pathways involved in AD, as brain regions are differentially affected by AD during the course of disease progression [12,14]. The hippocampus is one of the earliest brain regions to be affected and thus most studies have been aimed at this region. However, data from the other regions, especially those that are less affected by AD such as the cerebellum, are not well-studied and are sometimes even excluded from meta-analyses [15,16]. Exploring these changes in less affected brain regions may open new avenues to enhance the molecular understanding of AD pathogenesis and may reveal key disease mechanism in affected brain regions [17]. Therefore, in this study we have combined multiple gene expression datasets from five brain regions including the hippocampus, cerebellum, frontal, entorhinal and temporal cortices of AD patients and healthy controls, and used robust rank aggregation (RRA) meta-analysis to find robust differentially expressed genes (DEGs) between AD cases and healthy controls. We further investigated enriched pathways, related miRNAs and transcription factors of these DEGs.

## 2. Materials and Methods

### 2.1. Search Strategy

A comprehensive search was performed through the National Center for Biotechnology Information (NCBI) Gene Expression Omnibus (GEO) datasets (https://www.ncbi.nlm.nih.gov/geo/, accessed on 25 March 2021) to identify eligible data from inception to March 2021. The following key words were used: “Alzheimer”, “hippocampi”, “hippocampus”, “entorhinal”, “temporal”, “frontal” and “cerebellum”, then three filters including ‘Homo sapiens’, ‘Series’ and ‘Expression profiling by array’ were applied. In addition, references of all included studies were screened to find other relevant studies.

### 2.2. Dataset Selection

Datasets were included in our study if they met the following inclusion criteria: (1) dataset was original; (2) reported gene expression in hippocampus, cerebellum, frontal, entorhinal and temporal cortices of AD patients; (3) reported gene expression datasets for both cases and controls; (4) when the same authors published two or more datasets possibly using same data or re-analyzed pre-existing datasets, we used the most comprehensive dataset. GEO2R tool was used to analyze datasets and extract differentially expressed genes (DEGs) between AD cases and healthy controls. DEGs with adjusted *p*-value less than 0.05 were considered significant.

### 2.3. Integrated Genomic Analysis

Herein, we utilized the R package RRA to integrate microarray datasets downloaded from the GEO database and identify robust DEGs. The RRA method generates a relevant list from input lists even if they are incomplete. Robust DEGs with a Bonferroni-corrected *p*-value less than 0.05 were considered statistically significant.

### 2.4. Pathway Analysis

To identify relevant pathways, two lists of down- and up-regulated robust DEGs were submitted separately in Enrichr, a web-based tool for comprehensive gene set enrichment analysis (https://maayanlab.cloud/Enrichr/, accessed on 16 July 2021) [18]. KEGG (Kyoto Encyclopedia of Genes and Genomes) pathway was then used to find the enriched pathways from the submitted robust DEGs. Pathways with an adjusted *p*-value less than 0.05 were considered statistically significant.

### 2.5. Protein–Protein Interaction Analysis

Protein–protein interaction (PPI) networks were analyzed using Cytoscape string App plugin with the confidence score > 0.05 as previously described [19]. Briefly, differentially expressed genes were loaded into Cytoscape, and the *Homo sapiens* database in the StringDB was selected to reveal the protein interaction between differentially expressed proteins. In addition, hub genes within the protein network were identified using CytoHubba, a plugin within Cytoscape, based on the Maximal Clique Centrality (MCC) algorithm.

### 2.6. Gene–miRNA Interaction Analysis

The gene–miRNA interaction analysis was carried out in NetworkAnalyst (https://www.networkanalyst.ca/, accessed on 10 November 2021) [20], which uses collected data of validated miRNA-gene interaction from TarBase and miRTarBase [21,22]. Related miRNAs were obtained from both of these databases and common miRNAs were obtained using a Venn diagram analysis (http://bioinformatics.psb.ugent.be/webtools/Venn/, accessed on 10 November 2021). The miRNAs were then ranked using network topology measurements including degree and betweenness centrality, and the top five interactions were reported.

### 2.7. Gene-Transcription Factors Interaction Analysis

Gene-transcription factor interactions were discerned using NetworkAnalyst. Official gene symbols were submitted and related TFs were explored from three sources including Encyclopedia of DNA Elements (ENCODE) ChIP-seq data, ChIP Enrichment Analysis (ChEA) and the JASPAR database [23,24,25]. Common TFs between these three datasets were obtained using a Venn diagram analysis, and these common TFs were ranked based on network topology measurements including degree and betweenness centrality, and the top five interactions were reported.

## 3. Results

### 3.1. Search Results and Characteristics of Selected Studies

The initial search through GEO yielded 37 datasets, of which 10 datasets including 820 AD cases and 626 healthy controls met the eligibility criteria and were included in our study. The basic characteristics of the included studies are given in Table 1. There were seven datasets for the frontal cortex (GSE118553, GSE48350, GSE5281, GSE33000, GSE44770, GSE36980, GSE122063), five datasets for the temporal cortex (GSE118553, GSE5281, GSE36980, GSE122063, GSE132903), four datasets for the hippocampus (GSE48350, GSE5281, GSE36980, GSE29378), three datasets for the entorhinal cortex (GSE118553, GSE48350, GSE5281), and two datasets for the cerebellum (GSE118553, GSE44768).

**Table 1:** Characteristics of the selected datasets based on the criteria of this study.

Datasets	Country	Number of AD/CTR	Age (years) AD/CTR	Postmortem Interval (h) AD/CTR	Brain Region (s)	Reference
GSE118553	UK	52/27	82.9 ± 8.7/70.6 ± 15.9	39.9 ± 21.3/ 37.1 ± 20.7	Cerebellum/Entorhinal/Frontal/Temporal	[26]
GSE44768	USA	129/101	-	-	Cerebellum	[27]
GSE48350	USA	15/39	85.7 ± 6.3/64.8 ± 9.5	-	Entorhinal/Frontal/Hippocampus	[28]
GSE5281	USA	33/14	79.9 ± 6.9/79.8 ± 9.1	2.5/2.5	Entorhinal/Frontal/Temporal/Hippocampus	[29,30]
GSE33000	USA	310/157	80.6 ± 9.0/63.5 ± 9.9	13.7 ± 7.4/22.4 ± 5.8	Frontal	[31]
GSE44770	USA	129/101	-	-	Frontal	[27]
GSE36980	Japan	26/62	83.0 ± 5.7	-	Frontal/Temporal/Hippocampus	[32,33]
GSE122063	USA	12/11	80.9 ± 7.4/78.6 ± 8.5	8.0 ± 4.0/ 9.0 ± 3.0	Frontal/Temporal	[34]
GSE132903	USA	97/98	85.02 ± 6.75/84.98 ± 6.90	-	Temporal	[35]
GSE29378	USA	17/16	77.3 ± 9.1/81.7 ± 6.9	11.2 ± 6.3/10.8 ± 6.8	Hippocampus	[36]

### 3.2. Robust Differentially Expressed Genes

**Figure 1:**
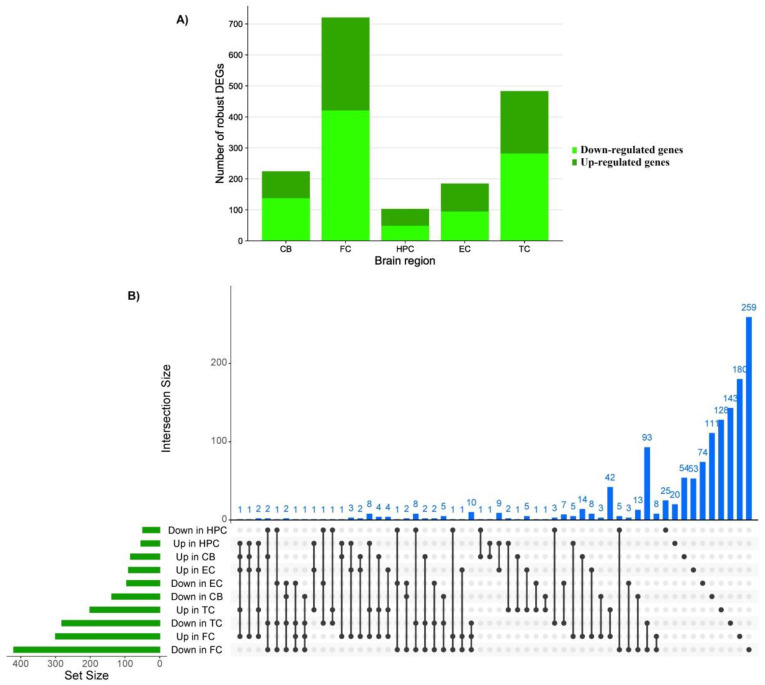
(**A**) Number of down and up-regulated robust genes in each brain region; (**B**) upset plot indicates the overlap of robust differentially expressed genes with either increased or decreased abundance in different brain regions; DEGs, differentially expressed genes; CB, cerebellum; FC, frontal cortex; HPC, hippocampus; EC, entorhinal cortex; TC, temporal cortex.

DEGs in each brain region were extracted from GEO using the GEO2R tool based on the limma R package (*p*-value < 0.05). In the next step the RRA R package was used to identify the robust DEGs [37]. This process identified a total of 1713 robust DEGs. The numbers of down- and up-regulated robust DEGs in each tissue were, respectively, 138 and 84 in the cerebellum, 420 and 300 in the frontal cortex, 49 and 54 in the hippocampus, 95 and 90 in the entorhinal cortex, and 282 and 201 in the temporal cortex. Interestingly, Serpin Family A Member 3 (*SERPINA3*) was up-regulated in all brain regions and none of the robust down-regulated genes were shared between all brain regions (Figure 1 and Supplementary File 1). The full list of robust DEGs and their scores can be found in the Supplementary File 1.

### 3.3. Pathways Enriched by Differentially Expressed Genes

**Figure 2:**
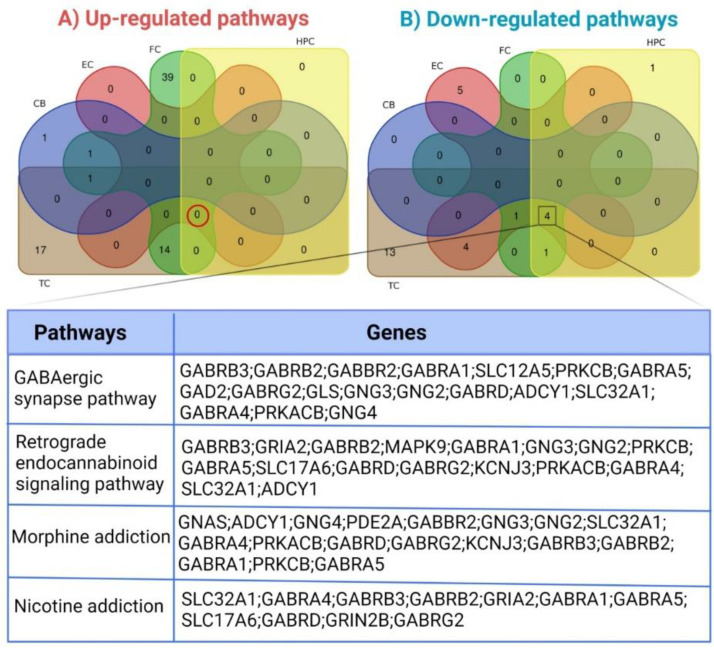
Venn diagram of KEGG pathway enrichment analysis on (**A**) Up-regulated and (**B**) Down-regulated pathways based on adjusted *p*-value. TC, temporal cortex; FC, frontal cortex; EC, entorhinal cortex; HPC, hippocampus; CB, cerebellum.

Enrichr and subsequently KEGG were used to predict pathways enriched separately by down- and up-regulated robust DEGs, and the pathways with an adjusted *p*-value of less than 0.05 were considered statistically significant. The top three enriched pathways in each brain region are provided in Table 2. Interestingly, based on adjusted *p*-value, the GABAergic synapse pathway, enriched by 18 robust DEGs, and the retrograde endocannabinoid signaling pathway, enriched by 17 robust DEGS, were meaningful pathways that were significantly down-regulated in all brain regions except the cerebellum (Figure 2), which is known to be less affected by AD. In addition, temporal and frontal cortices showed a higher number of altered pathways. A full list of enriched pathways for each brain region is provided in Supplementary File 2.

### 3.4. Protein-Protein Interaction Analysis

**Figure 3:**
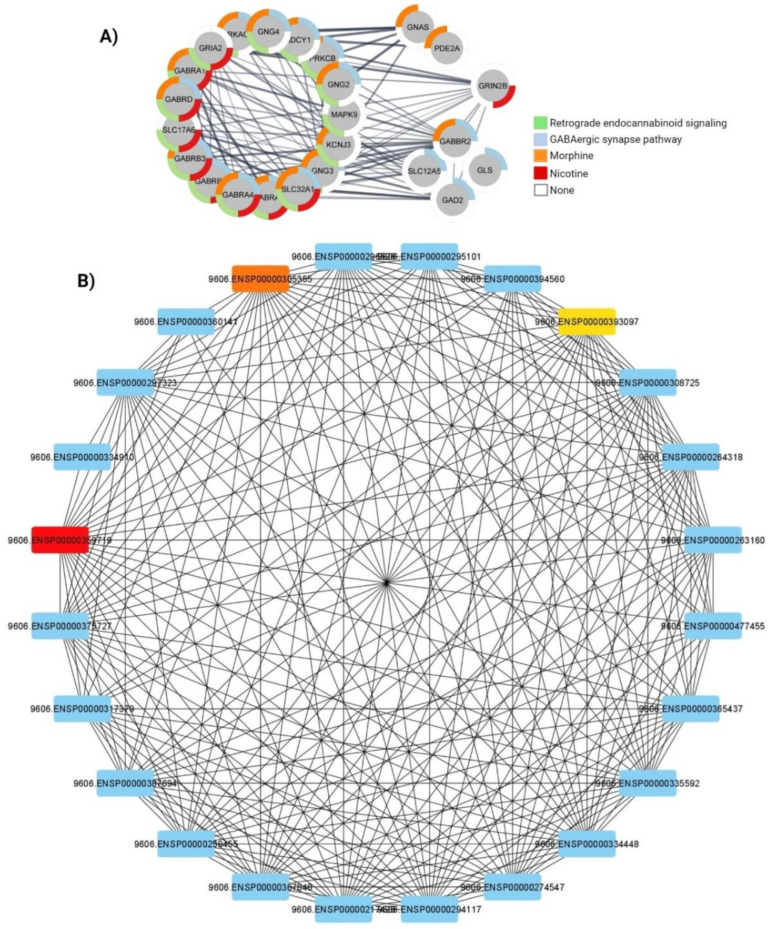
Functional interaction networks analyzed by the String Cytoscape plug-in. (**A**) PPI network of genes related to GABAergic synapse pathway (red), retrograde endocannabinoid signaling (green), morphine (blue) and nicotine (orange). (**B**) In addiction, *PRKACB* (red), *PRKCB* (orange) and *GABRA1* (yellow) found the top hub genes based on the MCC algorithm.

PPI network between down regulated genes in common pathways including GABAergic synapse pathway, retrograde endocannabinoid signaling, morphine and nicotine addiction-related pathways were retrieved using the Cytoscape stringApp plug-in. Furthermore *PRKACB*, *PRKCB* and *GABRA1* found as the top three hub genes through CytoHubba and based on the MCC algorithm (Figure 3).

### 3.5. Gene–miRNA Interaction and Targeting miRNA Analysis

**Figure 4:**
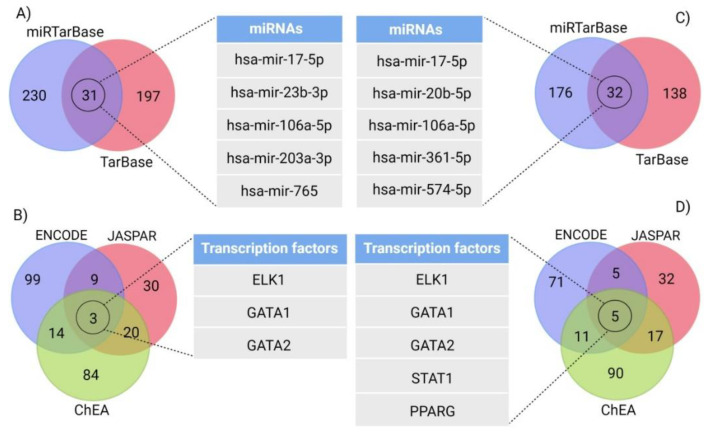
Transcription factors and miRNAs analyses. (**A**,**B**) results represent Venn diagram analysis for the top five miRNAs and the three TFs that interact with the robust DEGs of the GABAergic synapse pathway. (**C**,**D**) show Venn diagram analysis and the top five miRNAs and the TFs interacting with the robust DEGs involved in retrograde endocannabinoid signaling.

TarBase, miRTarBase and miRecords databases in NetworkAnalyst were used to indicate miRNAs interacting with the GABAergic synapse pathway and the retrograde endocannabinoid signaling pathway down-regulated genes, which returned 458 and 346 miRNAs respectively. There were 31 and 32 miRNAs common among all the databases for DEGs involved in the GABAergic synapse pathway and the retrograde endocannabinoid signaling pathway, respectively, and these were ranked based on network topology measurements including degree and betweenness centrality. The top five miRNAs are shown in Figure 4, and the top three miRNAs that were shared between the GABAergic synapse pathway and the retrograde endocannabinoid signaling pathway are provided in Table 3. 

### 3.6. Gene–Transcription Factors Interaction Analysis

Gene-transcription factors (TFs) interaction analysis was performed in NetworkAnalyst, in order to identify upstream regulators of the down-regulated robust DEGs involved in the GABAergic synapse pathway and the retrograde endocannabinoid signaling pathway. All transcription factors from the three different databases (ENCODE, ChEA and JASPAR) were extracted in NetworkAnalyst. Comparison of the identified TFs from these databases using a Venn diagram analysis yielded 3 shared TFs in the GABAergic synapse pathway and 5 TFs in the retrograde endocannabinoid signaling pathway (Figure 4). The top three TFs that were shared between the GABAergic synapse pathway and the retrograde endocannabinoid signaling pathway are shown in Table 3.

## 4. Discussion

In this study, ten microarray gene expression datasets available in the public domain were analyzed using an integrated genomic approach. The datasets comprised 1446 brain samples from five brain regions, including the hippocampus, cerebellum, frontal, entorhinal and temporal cortices. Between the AD cases and the healthy controls, 1713 robust DEGs were identified, showing different dysregulated pathways in these brain regions. The down-regulation of the GABAergic synapse pathway and the retrograde endocannabinoid signaling pathway was common among the hippocampus, frontal, entorhinal and temporal cortices, all of which have been shown to be more impacted by AD, compared to the cerebellum.

A balance between neural excitation and inhibition is vital to proper brain function and its disruption contributes to several neuronal disorders such as AD. The down-regulation of the GABAergic synapse pathway and the decreased levels of Gamma-aminobutyric acid (GABA) in the hippocampus, frontal, entorhinal and temporal cortices found in this study have also been reported in other human and mice AD studies [35,36]. GABA is the main inhibitory neurotransmitter within the mammalian central nervous system [38]. A decreased level of GABA receptor subunits such as *GABRA1, GABRA5, GABRB1, GABRB2, GABRB3, GABRG1, GABRG2* and *GABRG3* have been observed at the transcriptional and protein levels in the hippocampus of AD patients [39,40,41,42,43].

However, in contrast to these results, the expression of another GABA receptor named *GABRA6* was increased in cultured rat cerebellar granule neurons following treatment with amyloid beta (Aβ), one of the main pathological hallmark proteins implicated in AD [43,44]. Studies in both mice and humans have reported that the presence of Aβ can decrease the numbers and activity of GABA inhibitory interneurons, leading to impaired synaptic transmission and disrupted neural network activity, and resulting in cognitive impairment [45]. Interestingly, transplantation of GABA progenitor neurons into the hippocampus of mice overexpressing Aβ restored normal learning and memory [46].

Furthermore, the accumulation of phosphorylated tau (p-tau), another pathological hallmark protein of AD, in the hippocampus reduced extracellular GABA level and led to tau-induced anxiety in mice. In addition, p-tau accumulation in GABAergic interneurons in the dentate gyrus of AD patients and mice also caused disruption of GABAergic transmission [47,48]. This means that the GABAergic synapse pathway is down-regulated in AD, although there are some conflicting results from mice models [43]. However, AD has a complex neuropathological basis and mice models may not accurately represent underlying pathological events of AD in humans.

Another common down-regulated pathway between the AD sensitive brain regions was the retrograde endocannabinoid signaling pathway. The endocannabinoid system (ECS) is comprised of endocannabinoids (eCBs) that are endogenous lipid-based neurotransmitters, along with their receptors such as cannabinoid receptor 1 (CB1R) and cannabinoid receptor 2 (CB2R), and the enzymes that are involved in their synthesis and degradation [49]. Arachidonoyl ethanolamide (anandamide or AEA) and 2-arachidonoylglycerol (2-AG) are the two main endocannabinoids [49]. ECS plays a vital role in the central nervous system function and in the regulation of the endocrine and immune systems [50,51]. Endocannabinoid-mediated retrograde signaling was first reported in 2001, where eCBs were shown to mediate a type of short-term synaptic plasticity known as depolarization-induced suppression of inhibition (DSI)/excitation (DSE) [52,53]. Later, the effect of eCBs on long-term depression (eCB-LTD) on both excitatory and inhibitory synapses was also observed [54]. Although the alteration of ECS components in AD animal models has remained controversial and mostly unchanged levels of these components in the hippocampus have been reported, beneficial effects of CB1R activation via exogenous cannabinoids has been observed against Aβ-induced neurotoxicity, particularly microglia activation in several cell models [54,55,56]. Moreover, eCBs have anti-inflammatory and neuroprotective properties, and since neuroinflammation is a key driver of neurodegenerative diseases such as AD, restoration of dysregulated ECS has emerged as a promising therapeutic option [55].

Interestingly, the down-regulation of retrograde endocannabinoid signaling also has been observed in a meta-analysis of gene expression data from patients with mild cognitive impairment (MCI) [57]. MCI patients are at a higher risk of developing AD as MCI is considered an early stage of AD. Thus, the molecular changes in the respective brain regions of MCI patients may represent early changes that mediate the development of AD [57,58]. The down-regulation of the retrograde endocannabinoid system in MCI patients, and in the four AD sensitive brain regions but not in the cerebellum of AD patients, highlighted dysregulation of this pathway as an early and key change in AD development. As such, this can be a promising therapeutic target for early intervention against AD progression.

The miRNAs and TFs that interact with robust DEGs in the GABAergic synapse pathway and in retrograde endocannabinoid signaling were also investigated here. Among the identified miRNAs, hsa-mir-17-5p, hsa-mir-106a-5p and hsa-mir-373-3p were found to interact with robust DEGs of both pathways. Recently, the elevated levels of hsa-mir-17-5p have been reported in the human AD brain and the microglia of AD mice models, coupled with the down-regulation of autophagy markers, that consequently impaired Aβ clearance [59]. In contrast to our results, decreased serum level of hsa-mir-106a-5p has previously been observed in AD patients [60]. 

However, recent studies have reported that hsa-mir-106a-5p decreased the level of VEGFA, which plays a protective role against cognitive impairment in AD patients, and its level is decreased in AD patients [61,62]. Therefore, the increased level of hsa-mir-106a-5p in AD patients may be involved in cognitive impairment, and thus its abundance may be altered in patients with mild cognitive impairment. No data is yet available in the published literature on the role of hsa-mir-373-3p in AD; however, a recent study indicated that hsa-mir-373-3p inhibits epithelial–mesenchymal transition in choriocarcinoma through targeting *TGFβR2* [63]. Interestingly, the level of *TGFβR2* in AD patients was shown to be about 50% lower than normal [64], therefore hsa-mir-373-3p may be associated with decreased level of *TGFβR2* in AD patients. Collectively, there are limited data about these miRNAs and further studies are needed to establish their role in AD.

In order to investigate the upstream regulation of robust DEGs, TF analysis was performed. This returned the three TFs, ELK1, GATA1 and GATA2 that interact with robust DEGs involved in both the GABAergic synapse pathway and the retrograde endocannabinoid signaling pathway. ELK-1 belongs to the ternary complex factor (TCF) subfamily of ETS-domain TFs and its presence in diverse brain regions has been previously reported [65]. ELK-1 plays a dual role in neuronal function; while its expression is required for proper neuronal differentiation, overexpression and distinct phosphorylation of this TF is toxic for neuronal cells [66]. ELK-1 decreases expression of the presenilin 1 gene (PS1), which is involved in the proteolytic processing of the amyloid precursor protein (APP) and the generation of amyloidogenic Aβ, while, in turn, the presence of Aβ decreases ELK-1 activation and may derepress PS1 expression [66,67,68]. The other two identified TFs, GATA1 and GATA2, belong to the GATA family. Aberrant expression of GATA1 has been reported in AD patients previously, and has been shown to induce the expression of the β-amyloid precursor mRNA, and modulate γ-secretase activity. GATA1 may therefore be involved in the impairment of the synaptic plasticity and cognitive function [69,70]. GATA2, on the other hand, has been reported to play a key role in neuroglobin (NGB) activation, which has been shown to exhibit protective effects for neuronal cells in AD [71]. The expression of NGB is increased in early and moderate stages of AD but significantly decreased in advanced stages, which might be due to the decreased levels of GATA2 in the advanced stages of AD [72,73]. However, further studies are needed to evaluate this association. Interestingly, previous analysis of DEGs between the entorhinal cortex of AD patients and healthy controls also showed alteration of the retrograde endocannabinoid signaling pathway and predicted GATA1 and GATA2 as the upstream regulators of the identified DEGs in AD [74]. Our results here identified altered pathways in each brain region of AD patients and shared pathways between AD sensitive brain regions. Furthermore, our study also predicted miRNAs and TFs that interact with the DEGs involved in these shared pathways. However, experimental data about these regulators are limited and further studies are needed to reveal their role in AD pathogenesis.

## 5. Conclusions

In this study, we identified robust DEGs in different brain regions of AD patients and investigated enriched pathways using these genes. Our results suggested that the GABAergic synapse pathway and the retrograde endocannabinoid signaling pathway are two key pathways involved in AD pathogenesis, shared between all AD sensitive brain regions but not cerebellum. Further analyses revealed hsa-mir-17-5p, hsa-mir-106a-5p and hsa-mir-373-3p as potential miRNAs that may be involved in the down-regulation of these pathways. TF analysis further demonstrated ELK-1, GATA1 and GATA2 are upstream regulators of these pathways. Additional studies using large cohorts of AD patients are warranted to assess the potential therapeutic targets related to these pathways, and determine the miRNA and TFs involved in their regulation.

## Figures and Tables

**Table 2:** The top three enriched pathways by robust DEGs in each brain region, determined using Enrichr and KEGG.

Brain Region	Pathway	Alteration	*p*-Value	Adjusted *p*-Value	Odd Ratio	Combined Score	Genes
Cerebellum	Mineral absorption	Up-regulated	0.000005926	0.0007467	22.29	268.27	*MT2A, MT1A, MT1M, ATP2B4, MT1G*
	IL-17 signaling pathway	Up-regulated	0.00005312	0.003347	13.75	135.34	*NFKBIA, CEBPB, CXCL1, S100A9, S100A8*
	NF-kappa B signaling pathway	Up-regulated	0.01023	0.3936	7.09	32.49	*NFKBIA, GADD45A, CXCL1*
Frontal cortex	Neuroactive ligand-receptor interaction	Down-regulated	0.000001048	0.0002148	3.50	48.19	*GABBR2, GABRA1, CHRM3, EDN3, NPY5R, GABRA5, GABRA4, GRIK1, HTR2A, MCHR1, GABRG2, MCHR2, ADCYAP1, CORT, CCKBR, GLRB, SST, NMU, CRH, TAC3, TAC1, VIP, GABRD*
	GABAergic synapse	Down-regulated	0.000002641	0.0002252	6.71	86.16	*GABBR2, GABRA1, GNG3, GNG2, SLC32A1, GABRA5, GABRA4, GAD2, PRKACB, GABRD, GABRG2*
	Complement and coagulation cascades	Up-regulated	8.435 × 10^−15^	1.839 × 10^−12^	17.28	560.01	*C1QB, C1QA, C1S, CFH, C1R, C5AR1, CFI, F13A1, SERPINA5, C4B, C4A, C7, CFHR1, C3AR1, VSIG4, CFB, C1QC*
Hippocampus	Bacterial invasion of epithelial cells	Down-regulated	0.0003395	0.01724	13.26	105.91	*CDC42, ARPC1A, ARPC4, MET*
	Synaptic vesicle cycle	Down-regulated	0.0003567	0.01724	13.08	103.83	*ATP6V1G2, SLC32A1, SLC17A6, SLC1A6*
	Morphine addiction	Down-regulated	0.0006406	0.01724	11.12	81.74	*GABRA1, SLC32A1, GNG4, PDE2A*
Entorhinal cortex	Morphine addiction	Down-regulated	0.00008088	0.009868	12.52	118.00	*GABRB2, GABRA1, GNAS, ADCY1, GABRD*
	Gap junction	Down-regulated	0.0008877	0.02926	10.15	71.31	*GNAS, ADRB1, ADCY1, TUBB4A*
	GABAergic synapse	Down-regulated	0.0009261	0.02926	10.03	70.04	*GABRB2, GABRA1, ADCY1, GABRD*
Temporal cortex	GABAergic synapse	Down-regulated	4.244 × 10^−10^	9.295 × 10^−8^	12.30	265.54	*GABRB3, GABRB2, GABBR2, GABRA1, SLC12A5, PRKCB, GABRA5, GAD2, GABRG2, GLS, GNG3, GNG2, GABRD*
	Nicotine addiction	Down-regulated	4.089 × 10^−9^	4.478 × 10^−7^	20.63	398.46	*GABRB3, GABRB2, GRIA2, GABRA1, GABRA5, SLC17A6, GABRD, GRIN2B, GABRG2*
	Retrograde endocannabinoid signaling	Down-regulated	0.000001505	0.00007431	6.30	84.53	*GABRB3, GRIA2, GABRB2, MAPK9, GABRA1, GNG3, GNG2, PRKCB, GABRA5, SLC17A6, GABRD, GABRG2*

**Table 3:** The top three shared miRNAs and TFs between the GABAergic synapse pathway and the retrograde endocannabinoid signaling pathway; data were extracted from miRTarBase and JASPAR databases.

Name	Pathway	Degree	Betweenness
miRNA			
hsa-mir-17-5p	GABAergic synapse pathway	2	887.4
	Retrograde endocannabinoid signaling	3	1135.6
hsa-mir-106a-5p	GABAergic synapse pathway	2	887.4
	Retrograde endocannabinoid signaling	3	1135.96
hsa-mir-373-3p	GABAergic synapse pathway	1	0
	Retrograde endocannabinoid signaling	1	0
Transcription Factors			
ELK1	GABAergic synapse pathway	2	7.35
	Retrograde endocannabinoid signaling	2	8.13
GATA1	GABAergic synapse pathway	1	0
	Retrograde endocannabinoid signaling	1	0
GATA2	GABAergic synapse pathway	10	245.61
	Retrograde endocannabinoid signaling	10	261.51

## Supplementary Materials

The following supporting information can be downloaded at: https://www.mdpi.com/article/10.3390/cells11060987/s1, Supplementary File 1: RRA genes; Supplementary File 2: Enriched pathways.
